# Microfluidics by Additive Manufacturing for Wearable Biosensors: A Review

**DOI:** 10.3390/s20154236

**Published:** 2020-07-29

**Authors:** Mahshid Padash, Christian Enz, Sandro Carrara

**Affiliations:** 1 Laboratory of Integrated Circuits, École Polytechnique Fédérale de Lausanne, CH-2002 Neuchâtel, Switzerland or m.padash427@yahoo.com (M.P.); christian.enz@epfl.ch (C.E.); 2 Chemistry Department, Shahid Bahonar University of Kerman, Kerman 76169-13439, Iran

**Keywords:** wearable biosensors, metabolism, remote monitoring, sweat, microfluidic, 3D printing

## Abstract

Wearable devices are nowadays at the edge-front in both academic research as well as in industry, and several wearable devices have been already introduced in the market. One of the most recent advancements in wearable technologies for biosensing is in the area of the remote monitoring of human health by detection on-the-skin. However, almost all the wearable devices present in the market nowadays are still providing information not related to human ‘metabolites and/or disease’ biomarkers, excluding the well-known case of the continuous monitoring of glucose in diabetic patients. Moreover, even in this last case, the glycaemic level is acquired under-the-skin and not on-the-skin. On the other hand, it has been proven that human sweat is very rich in molecules and other biomarkers (e.g., ions), which makes sweat a quite interesting human liquid with regards to gathering medical information at the molecular level in a totally non-invasive manner. Of course, a proper collection of sweat as it is emerging on top of the skin is required to correctly convey such liquid to the molecular biosensors on board of the wearable system. Microfluidic systems have efficiently come to the aid of wearable sensors, in this case. These devices were originally built using methods such as photolithographic and chemical etching techniques with rigid materials. Nowadays, fabrication methods of microfluidic systems are moving towards three-dimensional (3D) printing methods. These methods overcome some of the limitations of the previous method, including expensiveness and non-flexibility. The 3D printing methods have a high speed and according to the application, can control the textures and mechanical properties of an object by using multiple materials in a cheaper way. Therefore, the aim of this paper is to review all the most recent advancements in the methods for 3D printing to fabricate wearable fluidics and provide a critical frame for the future developments of a wearable device for the remote monitoring of the human metabolism directly on-the-skin.

## 1. Introduction

Nowadays, the prevention of diseases by monitoring the early stages is considered a very cost-effective approach with respect to treatment costs once the diseases are fully manifested. This new approach also leads to better health outcomes [1,2]. In this endeavor, wearable biosensors have gained considerable attention. The high specificity, portability, fast detection, low-cost, and low-power features of biosensors have made them very suitable as wearable applications. Wearable devices have a considerable role in accomplishing these goals since the collection of crucial information in a continuous and non-invasive manner is easily obtained [3,4,5,6,7,8,9]. The USA announced 2015 as “the year of health care for wearables” [10], while The Huffington Post stated wearable technology is “the coming revolution in healthcare” [11]. Wearable biosensors are advancing toward non-invasive monitoring. In this regard, microfluidic systems are very effective and helpful. Due to the important role of microfluidics, manufacturing methods are required to be suitable for these goals. Therefore, an efficient, flexible, fast, and affordable manufacturing method plays a huge role in the future development of wearable biosensors and human health monitoring. Before we get into the manufacturing methods, first we will discuss the wearable technology, its application in health monitoring, and the role of microfluidics in their development.

**Figure 1:**
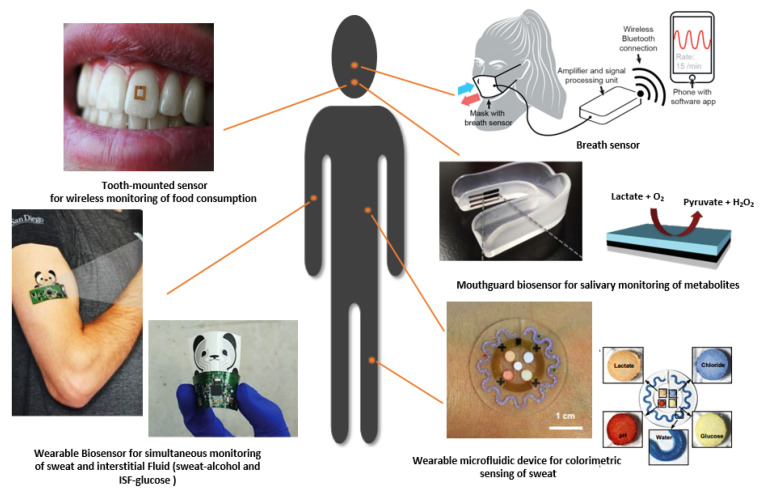
Different shapes of wearable devices for health monitoring (tooth-mounted sensor photo courtesy of Mike Silver, SilkLab, Tufts University, 23 October 2019 (reprinted with permission from [32,43,44,45])).

Wearable technology is often referring to a category of wearable gadgets that can be worn directly by a consumer for fun or just to track their physical activity and fitness. A different category of wearable technology is medical wearable devices that can be worn by patients on their skin, on different parts of the body, and often includes the tracking of the body’s physiological information related to health, in some cases, at molecular levels [12,13,14,15,16,17,18,19,20,21,22,23,24]. Wearable devices can collect data on a 24-h, seven-day basis, in several environmental settings, as people go through their daily routines at home or at work [25]. Wearable devices are able to relay physiological information as the body evolves over healthy and sick states. They can help persons to monitor themselves without expensive equipment, and neither educated professionals nor teams of expensive medical staff are required [15,26]. Moreover, the characterization of non-invasive and wearable technologies for diagnosis is extremely beneficial for both continuous health monitoring and diagnostics in early and pre-disease states. They also allow a quick access of clinical information by the patients, which encourages people to take more concern in their own health in a more comfortable and cheaper way, which also improves compliance [27,28]. In recent years, several wearable devices to gather the body’s physiological data have been proposed by the scientific literature, especially targeting personalized medicine and point-of-care diagnostics [29], as well as home and fitness monitoring. Wearable monitoring is provided by shirts [30], necklaces [31], tattoos [32], lenses [33], headbands [34], smart wristbands [35], watches [36], shoes [37], eyeglasses [38,39], wristbands, and patches [40,41]. Different kinds of wearable sensors perform clinical diagnostics by measuring the major electrolytes, metabolites, ions, acids, heavy metals, alcohols, and toxic gases directly acquired in different body fluids [25,42], as shown in Figure 1.

**Figure 2:**
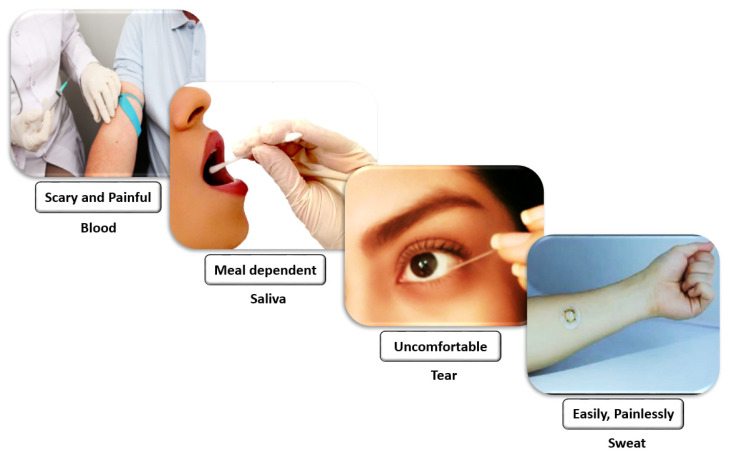
The methods of sampling for several candidate biofluids: Blood collection usually causes a bit of pain and can cause phobias and discomfort to patients. Before sampling saliva for a specific analysis, the time and content of the last meal should be considered. Tears can be uncomfortable or risky to sample. Sweat is easy to collect by painless techniques (reprinted with permission from [43], needle-professional-arm-human-body-blood-skin-647863, Photo by https://pxhere.com/en/photo/647863 is licensed under CC0 1.0, 5 October 2020).

There are several candidates as body fluids when looking to sample human molecules in different ways (Figure 2). Blood is the most widely exploited biological fluid for clinical diagnostics. Access to it is usually painful while difficult to reach with non-invasive techniques and more often impossible when trying with wearable platforms [26]. Its collection usually causes bit of pain, and it may also provoke phobias and cause discomfort to patients [28]. Its sampling is usually invasive and it is unsuitable for long-term continuous monitoring [42]. As an alternative to blood sampling, interstitial tissue has been considered, which is another option largely used by commercially available glucometers for diabetic patients. For the sampling of glucose from the interstitial tissues, we still need to typically pass through the skin, therefore, this approach is invasive as well. Blood is not necessarily informative for health monitoring in the cases of some metabolites, so it is possible to transition from blood to other body fluids, such as saliva, sweat, and tears for health monitoring. It provides non-invasive approaches and in-situ monitoring, which is more attractive for the long-term applications of the continuous monitoring of health in daily life [42].

Tears are a promising fluid for protein, lipids, and glucose detection. Tear sampling or its continuous monitoring can be uncomfortable or risky in terms of irritation, which can produce side-effects as well as mislead the sensor readings (e.g., by variations of pH). A capillary micropipette and swab are usually used for tear sampling. When they are being used, the eye usually reacts when coming close to an external object, and also some unwanted contact can cause irritation, which makes sampling uncomfortable. On the other, any irritation with increasing the production of tears can cause a reduction in the biomarker concentration. Saliva includes some markers for several diseases, such as cardiovascular diseases, oral and breast cancer, and human immunodeficiency virus (HIV) [46,47,48]. Because of the high alteration of the saliva composition from the last meal, it provides limited physiological insight. Instead, sweat is a promising fluid for wearable sensing, providing several analytes such as ions, alcohols, and drugs [15].

**Figure 3:**
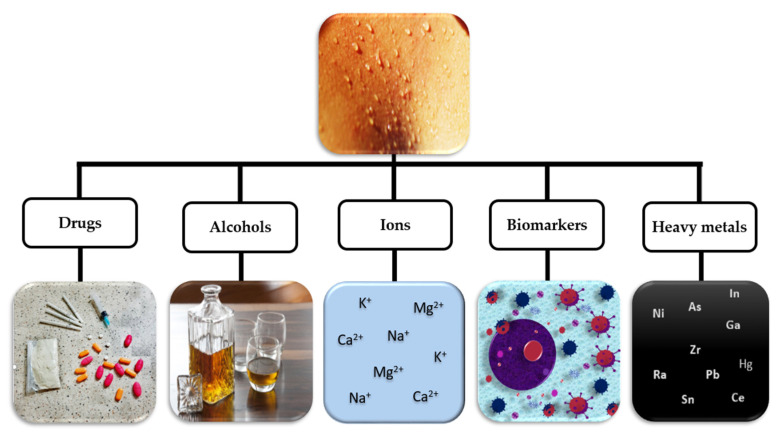
Different kinds of analyte in sweat: biomarkers, ions, alcohols, drugs, and heavy metals (Sweat_body_fitness_sport_fit_training_active_young-552041, Photo by https://pxhere.com/en/photo/552041 is licensed under CC0 1.0, Bottle of whisky and three glasses on wooden table, Photo by Lovely2912 form https://pxhere.com/en/photo/1593175 is licensed under CC0 1.0, 5 October 2020).

Sweat is particularly interesting for non-invasive biosensing because it is an abundant source of information on the inner physiological health, which can be determined from analytes, several of them with potential as biomarkers for diagnosing diseases, e.g., such as ions [49,50,51], alcohols [18,52], glucose [53,54], lactates [19,55] drugs, and heavy metals [15], as schematically shown in Figure 3.

**Figure 4:**
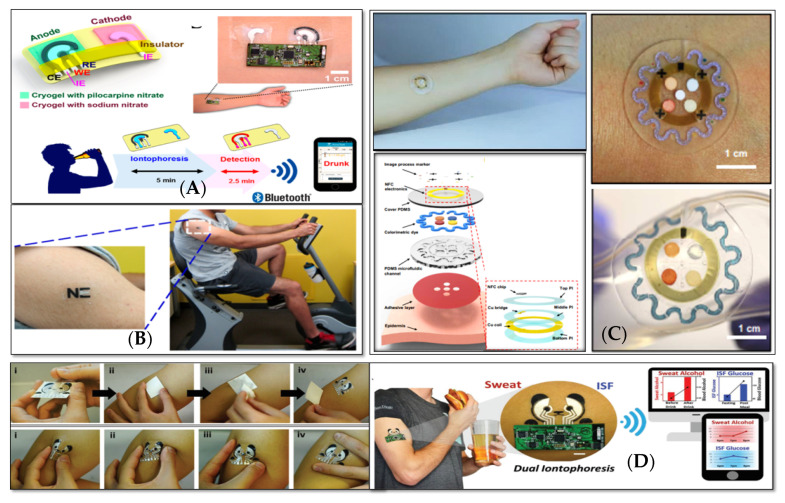
Wearable sweat analyzing platforms: (**A**) a wearable tattoo-based iontophoretic-biosensing system for alcohol monitoring (reprinted with permission from [18]), (**B**) an electrochemical tattoo biosensor for real-time non-invasive lactate monitoring in human perspiration (reprinted with permission from [19]), (**C**) a wearable microfluidic device for the capture, storage, and colorimetric sensing of sweat for markers such as chloride and hydronium ions, glucose, and lactate (reprinted with permission from [43]), and (**D**) a wearable biosensor platform for the simultaneous monitoring of sweat and interstitial fluid for components such as glucose and alcohol, subfigure i–iv shows the tattoo application process and mechanical deformation tests of transferred tattoo respectively (reprinted with permission from [32]).

Genomics and proteomics play an important role in searching for new biomarkers in sweat. For example, dermcidin (DCD) and prolactin inducible protein (PIP), in addition to blood, have been found in sweat too [56]. DCD and its receptors are present and overexpressed on the cell surfaces of invasive breast carcinomas, and their lymph node metastases as well as in brain neurons. PIP is instead overexpressed in prostate cancer and metastatic breast cancer [56]. Tozser and his group analyzed dermcidin (DCD) and prolactin inducible protein (PIP) in sweat by label-free mass spectrometry [57]. The discovery of new diagnostic molecules in sweat has, of course, pushed further the investigation of new wearable diagnostic devices, which can also be located close to the place of sweat generation, allowing a quick detection before the analytes biodegrade [15,28]. Therefore, and due to this new attention towards sweat analysis, several novel wearable and flexible sweat analyzing platforms have been recently proposed for in situ analyses and continuous health monitoring [12,16,18,19,43,53,54], as shown in Figure 4.

**Figure 5:**
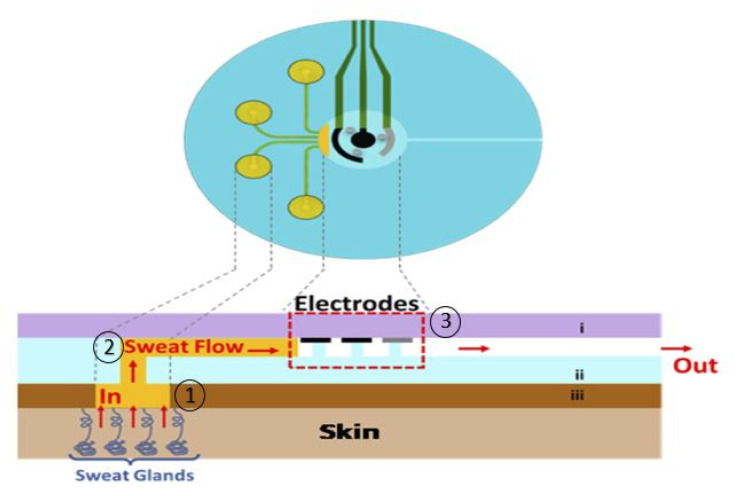
Schematic of wearable microfluidics: (**1**) sampling, (**2**) transferring to the site of detection, (**3**) detection by electrochemical sensors (reprinted with permission from [55]).

Despite the evident advantage in terms of non-invasive diagnostics, on-body sweat sensing presents several issues including but not limited to liquid evaporation, low sweat-volumes, irregular volumes in the case of non-stimulated sweating, contamination, interfering chemicals from the environment, the need for the continuous sampling of fresh sweat, and biodegradation [26]. An appropriate sweat sampling method is then very crucial to prevent measurement artefacts being evaporated and the contamination of sweat specimens. An effective sweat transport by a fast sampling system can minimize the crossover effect due to the mixing of new and old sweat samples. These undesirable effects are overcome by developing proper microfluidics [16,43]. Typically, the design of microfluidic platforms is carried out with the goal of improving the following functions: (1) collecting body fluids in proper ways, (2) transferring the body fluids to the detection site, and (3) supporting the detection in the right conditions [58]. Figure 5 shows a possible conceptual scheme of such a microfluidic system. Here, the microfluidics typically provides continuous sampling by conveying the sweat along a controlled fluidic channel and then enhancing the sensing in a well-defined encapsulated acquisition chamber. Thin microfluidic layers are typically designed as sweat collectors to bring the sweat on the electrodes, preventing any further re-absorption of the electrodes back to the skin, and also preventing sweat evaporation [59,60]. If built with soft and flexible materials, microfluidics easily addresses some of the requirements for wearable devices, such as being lightweight, comfortable, and conformable [58]. Moreover, the sensing accuracy and reliability is significantly improved by allowing the sampling of precise liquid amounts. This is particularly beneficial since body fluids are often secreted in limited quantities, while the lower sampling volume also reduces the burden on patients. Moreover, microfluidic systems may also integrate little reservoirs for solute storage in case of the need for further injections to the detection part, typically at controlled intervals, for detection purposes, e.g., in case of labelled detections [58]. The primary fabrication methods for microfluidics were photolithographic techniques and chemical etching with rigid materials, such as silicon and glass, which are non-flexible and expensive. Because of the limitations of these methods, three-dimensional (3D) printing methods were considered, which are cheaper and more flexible. In 3D printing processes, materials are solidified with computer control to create a three-dimensional object. In Section 2, we will take a brief look at microfluidics and the original methods of making them and their challenges. Finally, in Section 3 and Section 4, we will discuss why the traditional methods have given way to 3D printing methods today, and the details of the 3D printing methods suitable for wearable sensors and their applications.

## 2. Microfluidics

**Figure 6:**
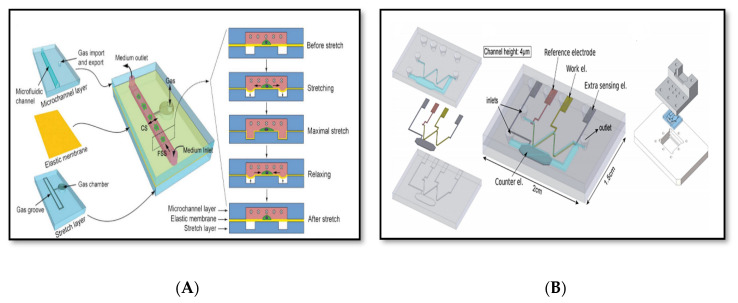
Microfluidic devices: (**A**) artificial organ-on-chip, (**B**) electrochemical detection of drugs (reprinted with permission from [68,69]).

Microfluidics is now a well-consolidated field of research, which deals with fabrication on the micron-scale of systems for manipulating fluids. It is most commonly identified by device fabrications with critical sizes of fluidic channels of less than 1 mm [61]. The field of microfluidics has grown rapidly over the last four decades since it emerged in early 1980s [62]. Numerous theoretical studies have been conducted in this field that play an important role in the making of an efficient product [63,64,65]. In one of these works, a novel continuous flow magnetophoretic microfluidic device for the separation of magnetic microparticles, based on size, is presented [64]. Some of the effective parameters on the motion of microparticles into the microchannel and the associated performance metrics are: the location of the outlets, microchannel height, fluid velocity, and ratio of inlet and of the outlet flow rates. In order to investigate the relationship between the operating and geometric parameters on device performance, a mathematical model is developed. Designers using a mathematical model can choose the parameters for the magnetophoretic microfluidic device with the best performance metrics. In recent years, many of the applications of microfluidic technologies to chemistry, biology, and medicine (e.g., see Figure 6) successfully appeared in the literature mainly due to their great advantages in terms of low volumes, high sensitivity, rapid processing, high spatial resolution, high integration with sensing components, and easy control. Undoubtedly, a short fabrication time, easy prototyping, and simple and cheap methods played an important role for the success of microfluidics [62,66,67].

One of the very first microfluidic devices was developed by Andreas Manz and his team in the early 1990s [70]. Such devices were originally fabricated in silicon or glass by a conventional, planar photolithographic technique, and chemical etching, by adapting techniques typically used in microelectronics. These kinds of methods are precise but expensive, non-flexible, and poorly suited to exploratory work by prototyping [71]. As the field has progressed, alternative methods, such as laminate manufacturing methods, various polymer molding technologies (e.g., hot embossing and injection molding) and 3D printing, have emerged for the fabrication of channels with the requisite sizes [61]. Traditional methods have failed to address some of the barriers, while the commercialization of microfluidic devices with 3D printing is able to overcome said barriers. Some of these barriers include a non-standard user interface, complex control system, and the speed and cost of the liquid polymer (i.e., poly(dimethylsiloxane) (PMDS)) modeling. In the 3D printing method, various materials with different properties (e.g., transparent and biocompatible) have been developed that can be used according to the application of the microfluidic device. So, among the several possible methods, this review focuses on additive manufacturing, namely the modern industrial method to create three-dimensional objects, typically in a computer-controlled manner, by progressively adding material, typically in a layer-by-layer approach: the so-called 3D printing.

## 3. Three-Dimensional Printing Methods

**Figure 7:**
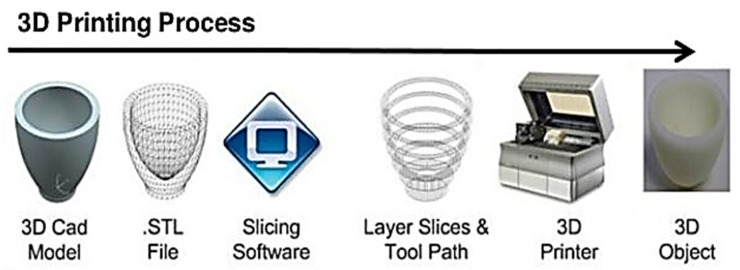
Three-dimensional printing process (3d-printing-a-2014-horizonwatching-trend-summary-report-9-638 by Kholoudabdolqader is licensed under CC-BY-SA-4.0, 1 October 2020).

Additive manufacturing, namely 3D printing, has enormous potential for a considerable contribution to the field of microfluidics. In particular, its ability to create truly three-dimensional structures with very complex features in a single step and start from a digital model has obvious attractions for the easy prototyping of very complex microfluidics [72]. This technology was developed originally by Charles Hull in 1986 [73]. In general, 3D printing is an additive manufacturing (AM) technique, proposed for the fabrication of a wide range of structures with computer-controlled processes based on three-dimensional (3D) digital models of the object to print (Figure 7). The process includes printing consecutive layers of materials that are formed on top of each other [73], while digital models provide an extremely flexible way to design and shape the objects. Typical fabrication thicknesses are in the range from 0.001 to 0.1 inches for each printed layer [74]. Additive manufacturing, or 3D printing as it is more often known, has received considerable interest in more recent years in both the academic community and the business society, and it has been mentioned as a third industrial revolution [72,75]. In fact, 3D printing offers many advantages with respect to traditional manufacturing, including an improved versatility, less waste, more freedom in design, a low-cost fabrication, high-automation, and short fabrication cycle time [73,76]. Three-dimensional printing technology allows for the creation of objects with complex internal structures with fewer space requirements [76]. Three-dimensional printing is also suitable for fabricating parts of various sizes from the micro- to macro-scale [73,75].

Product customization has been a challenge for traditional manufacturers, typically due to the high costs in fabricating the mold, especially for small-scale productions of custom-tailored products. On the other hand, 3D printing is able to print small quantities of customized products in plastic (in 3D) with extremely low costs compared to traditional mold-based productions. This is specifically useful in biomedical fields, whereby unique patient-customized products are also required [73].

**Figure 8:**
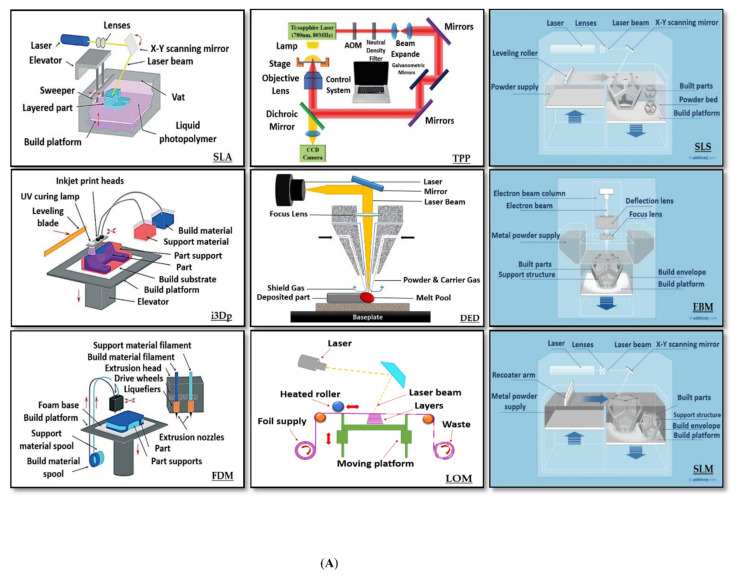

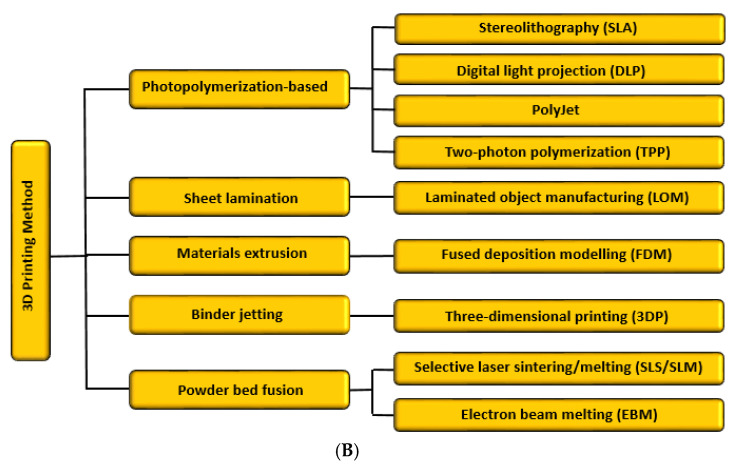
Three-dimensional printing methods: (**A**) various methods for additive manufacturing: stereolithography (SLA), two photon polymerization (TPP), selective laser sintering (SLS), multi-jet modeling (photopolymer inkjet printing (MJM))/ inkjet 3D printing (i3Dp), direct energy deposition (DED), electron beam melting (EBM), fused deposition modeling (FDM), laminated object manufacturing (LOM), selective laser melting (SLM), (**B**) related taxonomy on the base of the product printing way (reprinted with permission from www.additively.com, [77,78]).

Actually, the name “3D printing” includes various methods for additive manufacturing (see Figure 8).

By following the new incoming needs of human monitoring (e.g., serious critical concerns about the increasing medical costs related to the aging of the populations in west-countries), an emerging trend in mobile health (mHealth) is about developing new kinds of monitoring systems by the integration of different sensory devices in wearable single-platforms to achieve more complex and efficient monitoring functions for the new concept of digital biomarkers [79]. Of course, several new wearable devices are already on the market with the capability to acquire data, e.g., about temperature, location, physical activity, etc., while very few of these devices already on the market are capable of providing real measurements on molecules that are usually used in diagnostics and possible to find on-the-skin. On the other hand, many examples of possible approaches to realize wearable systems for molecular measurements have already appeared in the scientific literature. These kind of devices typically imply the intimate integration of electrochemical or optical biosensors with proper microfluidics in order to collect sweat from the skin and convey it, as well as its molecular content, to the sensory platform. Among the many different and possible approaches for fabricating such systems, we will focus here on 3D printing techniques and related materials for building wearable microfluidics to be then integrated in biosensors.

All 3D printing techniques are not appropriate for microfluidics. The most widely used 3D printing techniques for microfluidics are selective laser sintering (SLS), fused deposition modeling (FDM), inkjet 3D printing (i3Dp), laminated object manufacturing (LOM), two photon polymerization (TPP) and stereolithography (SLA) [78,80,81,82,83,84,85,86]. Since we are pointing out here the work done for wearable biosensors, this review focuses only on progress made towards the use of 3D printing for the fabrication of flexible microfluidics. Polymeric substrates are widely used in flexible devices [58]. The polymeric suitable approaches that have been already successfully to this aim include fused deposition modeling (FDM), inkjet 3D printing (i3Dp) and stereolithography (SLA) [72]. These 3D printing techniques also provide the opportunity for using multi-material to improve the quality of the final printed product [87,88]. Therefore, these three methods for 3D printing of wearable fluidics are discussed more in detail in the following sections of this paper. Table 1 summarizes the materials, benefits, and limitations of the three 3D printing methods suitable for wearable microfluidics.

### 3.1. Fused Deposition Modeling (FDM)

**Figure 9:**
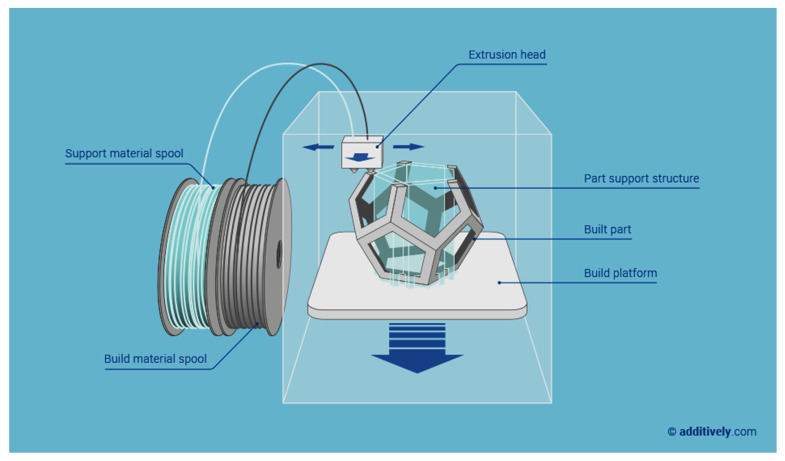
Schematic representation of FDM (reprinted with permission from www.additively.com).

The fused deposition modeling (FDM) appeared originally in a patent obtained in 1992 and this technology was, and still it is, commercialized by the company Stratasys, funded by Scott Crump, the inventor of this 3D printing approach [76]. This process works by depositing layers through extrusion and fast condensation, in different locations, driven by a computer based on a digital model of the object-to-build [73,89], as schematically shown in Figure 9. The method uses a motor-driven nozzle that moves in three dimensions. A continuous filament of a thermoplastic polymer is extruded on the build platform after heating the nozzle head. After extrusion, the material cools down and solidifies immediately.

In this method, the thermoplasticity of the polymer is strictly required to obtain the fused filament, which is a key component of the entire fabrication process. Thanks to this property, different polymer filaments may fuse together during printing and then solidify [73]. The mechanical properties of the printed parts are clearly dependent on the processing parameters. The main processing parameters are the width and orientation of the filaments, layer thickness, and air gaps in some of the layers or between the layers [73]. FDM can be successfully used in several applications with inexpensive biocompatible polymers such as polycaprolactone (PCL), polylactic acid (PLA), polybutylene terephthalate (PBT), and polyglycolic acid (PGA) [63,78]. The main benefits of FDM are the low-cost, high speed, and simplicity of the method [73].

Despite the advantages, FDM presents some drawbacks, such as the weak mechanical properties of the final realized solid and evident appearance of the layer-by-layer built structure, a poor surface quality [62], leakage due to the filament bonding when used for fluidics [89], and a limited number of thermoplastic materials are available to use [73]. The main cause of mechanical weakness is related to the inter-layer distortion [73]. Moreover, FDM usually requires support structures, of which removal may be difficult due to the complex internal features of the object [89]. Another drawback of FDM in producing microfluidic systems is the difficulty in appreciating the details of the printed systems. This drawback is because of light diffusion. Optical microscopy is used for the investigation of the systems. The polymers used for layering create light diffusion and make the optical investigation of the realized channels difficult [89]. To solve this problem, the optical transparent windows have been integrated inside the 3D printed microfluidic system [89]. Integrating optical transparent windows is typically possible by stopping the printing process to insert more transparent materials, and then restarting again the printing process. So, this method makes it hard to ensure a leakage-free sealing between the inserted part and the main body of the device [89]. Finally, the resolution of FDM is still on the way for improvement in terms of dealing with fluidics: it is less efficient than other 3D printing systems as compared, for example, to stereolithography (SLA). However, as we will see in the following, SLA presents higher costs for buying both the printer and the materials, while it requires more complex steps to complete a printed device [89]. On the other hand, the fabrication of microchannels with FDM is still a true challenge since the typical required size of a typical channel is usually smaller than the size of the extruded filaments [78]. Fabrications of channels with a well-defined sidewall geometry and straight walls are also difficult to obtain since the process creates rough surfaces [81]. After the extrusion process, the filaments cannot be arbitrarily joined at the channel intersections [62]. Because of the fast hardening of the extruded materials, the adjoining layers are not well fused, causing the low structural strength of FDM-printed products [78]. To increase the intra-layer strength, improvements such as creating covalent bonds for cross-linking between layers by using the thermally reversible Diels–Alder reaction and gamma-irradiation post-printing have been recently proposed for improving the properties of FDM-printed products [78]. or all these several reasons, the FDM remains a challenging technique for the fabrication of microfluidics.

**Figure 10:**
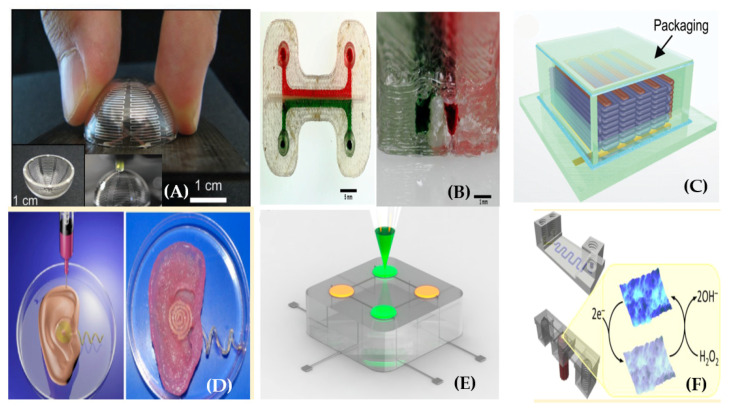
Different wearable devices created with FDM: (**A**) small antenna, (**B**) microfluidic device with an integrated membrane and embedded reagents, (**C**) inter-digitated Li-Ion micro-battery architectures, (**D**) bionic ears, (**E**) quantum dot light-emitting diodes, and (**F**) electrochemical detector (reprinted with permission from [88,90,92,93,95,98]).

On the other hand, we can successfully use the FDM method to fabricate some other components or parts usually required on integrated wearable systems: e.g., batteries [90], strain sensors on flexible substrates [91], light-emitting diodes (LEDs) [92], antennas on 3D surfaces [93], interconnects [94], electrodes within biological tissue [95], microfluidic pumps for wearable biomedical applications [96], electrochemical detectors [97] and other microfluidic devices [28,88], as shown in Figure 10.

For biomedical applications, inexpensive and biocompatible polymers from spools of filament are possible to be used in FDM [78,80,82]. In particular, the ones mainly used are: acrylonitrile butadiene styrene (ABS, the polymer of Lego), poly (lactic acid (PLA, a biodegradable polymer)), polycarbonate (PC), polyamide, polyethylene terephthalate (PET) polycaprolactone (PCL), polybutylene terephthalate (PBT), polyglycolic acid (PGA), polypropylene (PP), acetoxy silicone polymer and polystyrene (PS). There is an alternative version of FDM that uses, instead, liquid precursors which are extruded through a nozzle without heating. In this way, FDM may extrude a wide range of other materials, such as metallic solutions [80], hydrogels, and cell-based solutions, also including composites to strengthen the mechanical properties of 3D printed objects [73].

### 3.2. Inkjet 3D Printing (i3Dp)

**Figure 11:**
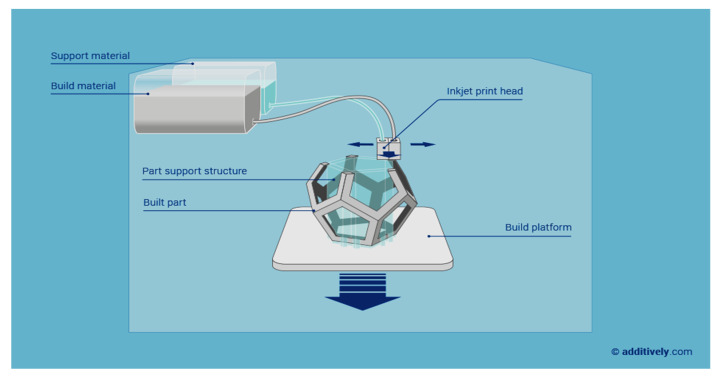
Schematic representation of inkjet 3D printing (i3Dp (reprinted with permission from www.additively.com)).

Inkjet printing is one of the main methods for additive manufacturing, especially used for printing ceramics. In this method, a nozzle and a stable ceramic suspension (such as zirconium oxide powder in water) are used. Via the injection nozzle, the suspension is pumped and deposited in the form of droplets onto the substrate (Figure 11). Then, the droplets form a continuous pattern that solidifies to hold further layers of printed materials. Several factors determine the quality of inkjet-printed objects: the solid content, particle size distribution of ceramics, nozzle size, viscosity of the ink, extrusion rate, and speed of printing [73].

There are two main types of ceramic inks: wax-based inks, and liquid suspensions. Wax-based inks are melted and deposited onto a cold substrate to solidify, while liquid suspensions are solidified by liquid evaporation [73]. Inkjet technology operates in the i3Dp process either in continuous or in drop-on-demand (DoD) mode [72]. Typically, the inks used in the continuous mode have a lower viscosity that provides a higher drop speed than those typically used for the DoD mode. The DoD mode generates smaller droplets with a higher placement accuracy. Therefore, the DoD is the better choice for 3D microfabrications and leads to finer and more repeatable microfluidic structures [72]. In the DoD technique, the pulse is piezoelectrically or thermoelectrically provided. In the piezoelectric DoD, the deformation of a piezoelectric element generates acoustic pulses. These pulses push droplets of ink from the nozzle. In the thermal DoD, a vapor blob is formed by heating the ink locally and then it is ejected as an ink droplet. In this case, the solvents must be volatile while in piezoelectric DoD, a large variety of organic solvents such as chloroform, dimethyl sulfoxide, and dimethylacetamide can be used.

**Figure 12:**
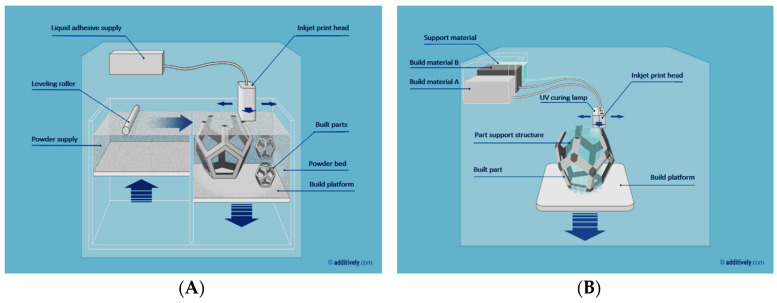
Inkjet 3D printing is split into two further categories: (**A**) powder-based, and (**B**) photopolymer-based (reprinted with permission from www.additively.com).

Inkjet 3D printing is further split into two categories (Figure 12): powder-based and photopolymer-based. In powder-based i3Dp, the powder particles are bonded with a polymeric sticking solution. In this process, a roller initially deposits a layer of ceramic powder, which is spread uniformly on the building stage. Then, the multi-channel printer head sprays droplets of adhesive onto the powder bed at the targeted areas. After completing the first layer, the building platform drops a second powder layer and bounding restarts again with the successive printing of adhesive. This process is repeated until the 3D object is formed. Once the process is completed, the printed object is typically surrounded by a feeble supporting powder that can be easily removed without further post-processing steps. Supporting powders are usually made by a mixture of gypsum, polymer, and silica particles, with adhesives that are composed by glycerol and water-soluble acrylates. These materials are recyclable. So, recycling the unused powder can further lower the costs of printing [72]. The resolution of these kind of printers is determined by some parameters such as the packing density, shape of the printed objects, and particle sizes of the used ceramic. Un-bound particles may increase the surface roughness and reduce the transparency of the printed object. These un-bound particles scatter the light, preventing the microscopy investigations usually required for investigating the printed microfluidics [72]. In the second kind of i3Dp (photopolymer-based), the head deposits small drops for both the build and support materials to create the object in a layer-by-layer process again. The typical materials used are acrylate photopolymers, starting from monomers, oligomers, or photo-initiators, then each printed layer is treated with an ultraviolet (UV) source. At least 100 different composite materials are available in the market, as further developed by a core of 17 primary photopolymers only [72].

Theoretically, an XY-resolution of 42 μm and a Z resolution of 16 μm is possible for a high resolution inkjet printing of 600 × 600 dpi [4]. In the case of mass production, a relatively inexpensive alternative way of inkjet printing is also possible for fabricating paper-based microfluidics [99]. Distinct advantages of i3Dp are the high resolution, simplicity, and low-cost [80,99]. This method is fast and efficient for easily printing quite complex structures with a high resolution by avoiding the use of lithography [73]. Inkjet 3D printing (i3Dp) easily modulates the shape and dimensions of the pattern and does not require the use of support structures [15].

With i3Dp, we can also realize the flexible resistive components and sensors by using conductive liquid metals [100]. Although the price is typically higher than that for FDM, i3Dp is probably the most commercially viable 3D printing approach for microfluidics [72]. This method creates microfluidic structures (also flexible) with precise control and manipulation of fluids that are small enough for the intended aim: typically, sub-millimeter or micrometer scales [83]. With this technique, vertically aligned channels usually have size stability and a smooth surface [72]. Interestingly, microfluidics can be realized directly on top of other systems, e.g., transducers or electrodes, without the need of any bonding or assembling steps [100]. In i3Dp, only inks containing weak organic solvents are acceptable [88], since strong organic solvents and hydrophobic materials, such as hexane, heptane, and toluene, damage the cartridges and other components of the printer. So, the main advantages of this method are the absence of sticking agents in between layers, protecting workability, and layer-by-layer fine structures [73]. On the other hand, some disadvantages are the quite high costs from changing materials during printing, removal of the support material (difficult for fully closed structures), and the drying of inks with consequent holes clogging in the print cartridges if the i3Dp system is not regularly used [72].

**Figure 13:**
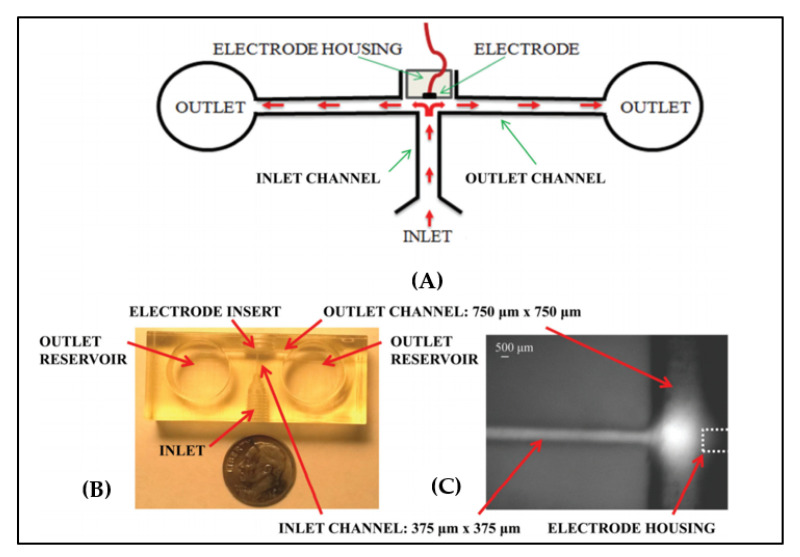
Wearable microfluidics created with i3Dp with a wall-jet electrochemical (WJE) configuration: (**A**) schematic of the WJE device, (**B**) top view of the 3D printed WJE device, (**C**) micrograph of a fluorescein plug hitting the electrode in the WJE design and flowing away from the electrode (reprinted with permission from [105]).

After the first microfluidic device proposed with i3Dp by McDonald and co-workers in 2002 [72], the development of accurate printers has been extensively improved to fabricate milli- and microfluidics [81]. As well as FDM, i3Dp is also regularly used to print biomedical systems other than wearable biosensors: for example, scaffolds for tissue engineering [73], orthopedic prosthesis [86], cardiac parts [101], and components for intracranial aneurysm surgeries [102]. In microfluidics, devices for flexible, planar, and multilayer microfluidics, membrane, on-chip gel electrophoresis, and wall-jet electrochemical detectors have been published so far [97,100,103,104,105], as shown in Figure 13.

Photopolymer i3Dp is also known by the names of PolyJet or multi-jet modeling (MJM). In MJM, a large range of photopolymers are used, including soft elastomers, liquid metals (i.e., EGaIn), acrylonitrile butadiene styrene (ABS), polystyrene (PS), polypropylene (PP), polymethylmethacrylate (PMMA), polycarbonate (PC), ethylene propylene diene monomer (EPDM), and high-impact polystyrene (HIPS) [72,80].

### 3.3. Stereolithography (SLA)

Stereolithography (SLA) was proposed as method of additive manufacturing in 1986 by Chuck Hull. He defined SLA as “a method and apparatus for making solid objects by successively ‘printing’ with thin layers of a curable material, e.g., a UV curable material, one on top of the other”. SLA was commercialized in 1988 and became the first commercialized 3D printing system ever proposed [72,82]. SLA uses an energy source (e.g., light or electron beams) for activation (radicalization) of the monomers (mainly acrylic or epoxy-based) in order to obtain polymer chains. After the polymerization, a pattern inside the resin layer is solidified to hold the next layer formation. After the full printing, the unreacted resin is then removed. In some cases, the printed parts need some post-process treatments, such as heating or photo-curing, in order to reach the desired mechanical performance [73]. While Hull described SLA only for material curable by UV, recent advances in resin photochemistry and laser technology achieved polymerization with modern high-intensity lasers or focused LED light sources in the visible wavelength range, using suitable types of photo-initiators [78,80].

**Figure 14:**
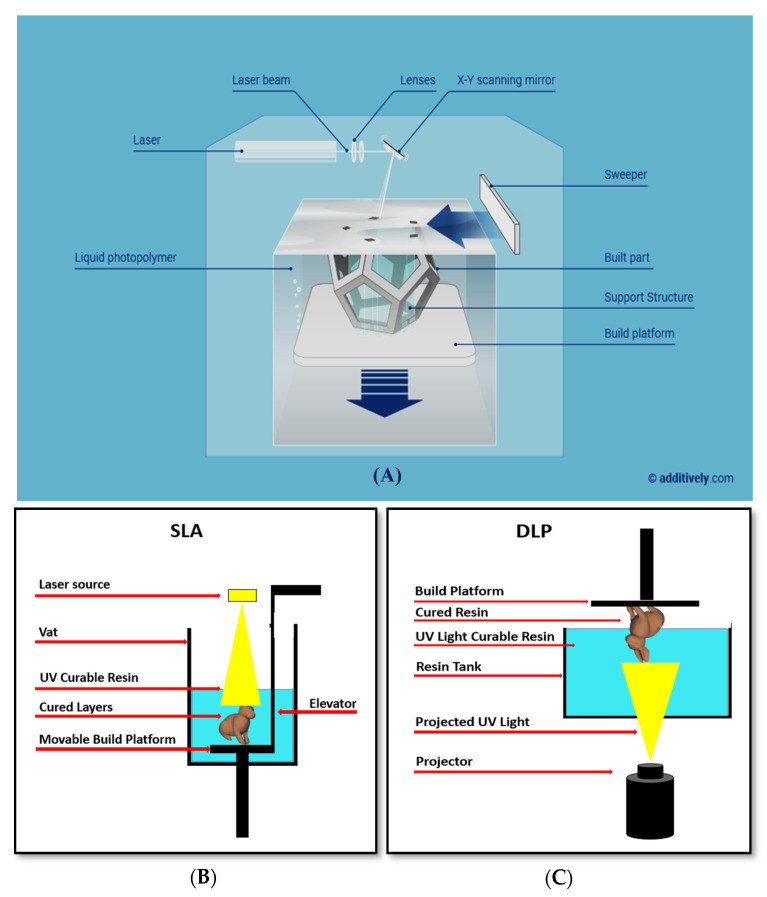
(**A**) stereolithography (SLA): (**B**) the bath, and (**C**) the bat configurations (reprinted with permission from www.additively.com).

In using SLA, the laser spot-size, the pixel resolution, the type and viscosity of the resin must be carefully considered for fabricating microchannels with the minimum cross-sectional area [78]. The thickness of each layer is affected by the energy of the light source and by the exposure time [73]. The laser spot-size and absorption spectra of the photoresins affect the resolution too [78]. SLA is possible in the two most important configurations: the free surface approach (bath configuration), and constrained surface approach (bat configuration). In both the cases, objects are formed by a liquid resin photopolymerized with either a scanning laser or a digital light projector (DLP) by a spatially controlled photopolymerization [72], as schematically shown in Figure 14. In the bath configuration, the vat depth limits the object height, while this limitation does not exist in the bat configuration. The time of curing is faster in the bat configuration because oxygen inhibits the process of photopolymerization, and the reaction happens far from the air–resin interface. For modifications in the constrained surface technique, the bottom plate is made sensitive to oxygen by a controlled oxygen inhibition to the last cured resin layer [78]. The bath configuration is the classical setup for SLA. In this configuration, a substrate is submerged in a tank of photoactive resin and a UV beam affects a two-dimensional (2D) cross-section onto the substrate when the resin polymerizes under illumination. After the completion of the 2D cross-section, the next step is the lowering of the substrate further into the resin by a predefined distance. Then, the next layer is polymerized on top of the previous layer by the UV beam. Working that way ensures that the focus of the UV beam does not change. A blade levels the surface with a further layer of uniform resin before the next step of exposure to UV light. As written, the ‘bat’ configuration is the name of the constrained surface approach. The reason for choosing this name is the fact that, as shown in Figure 14, the object is created hanging from a movable substrate like a bat from a ceiling. The movable substrate is hanging above the resin tank. The tank has an optically clear bottom and a non-sticking surface, so the printed structure does not stick to the substrate. The light source is typically located under the tank (picture on Figure 14B). The action of gravity on the forming surface, which rests for a certain settling time, refreshes and smooths the surface of the illuminated resin. The object is drawn out of the resin, rather than submersed in it, so only small amounts of resin with low viscosity are needed. Since the illuminated layer is not exposed to the atmosphere, oxygen inhibition is limited. Here, the height of the printed objects is not limited with respect to the bath configuration, and the bat configuration requires minimum cleaning steps. The cured layer is sandwiched between the resin vat and the previous layer. Sometimes, the solidified material strongly sticks to the bottom of vat. In these cases, the object may, unfortunately, break or deform when coming up from the vat [72].

SLA produces high-quality objects with a fine resolution down to 10 μm [73], and offers a good balance between the resolution, price, and performance [72]. The fabrication of micro- and nanostructures in a wide variety of shapes is possible as well [83]. There are a considerable number of different choices regarding the material properties, especially regarding the increasing range of the extrusion filaments: it is possible to also use conducting, flexible, and magnetic filaments, as well as a range of different colored polymers [84]. Materials like elastomers and ceramics are also allowed, as well as photoactive resins such as acrylate, clear acrylic, epoxy, hybrid resins and composites of different photopolymers [58,81]. Dispersions of ceramic particles are used to print ceramic–polymer composites or polymer-derived ceramifiable monomers, such as silicon oxycarbide [73]. Recently, Gong et al. investigated the effect of the optical property of SLA’s resin on the channel sizes in microfluidic systems. They found that there is fundamental exchange between the critical dose that penetrates into a flow channel during fabrication and the homogeneity of the optical dose within individual layers. In order to obtain a minimum channel size of 60 μm × 108 μm by 10 μm building-layers, they increased the resin absorbance and the XY plane resolution of the DLP illumination [72]. Transparent biocompatible resins are available too for SLA, so the realized microfluidics allow for the characterization of the internal fluid flow as well as the direct observation of the in-situ formation of droplets [106]. Other advantages are the smooth surfaces, custom low-cost resins, lack of need for external alignment, monolithic structures, and direct printing of the fluidic channels [84,107,108].

**Figure 15:**
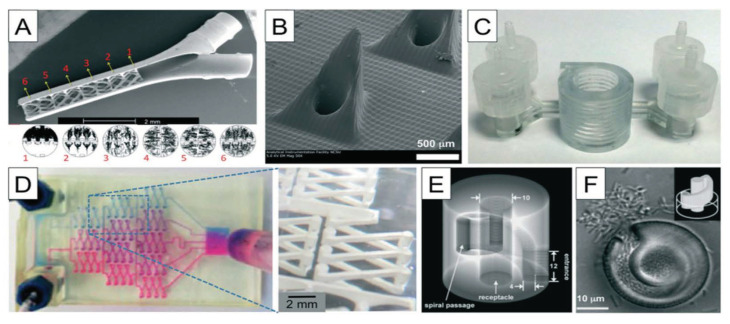
Examples of structures fabricated with SLA: (**A**) a micro-mixer, (**B**) hollow micro-needles, (**C**) Spiral microchannels for size-selective separation of bacterial cells, (**D**) gradient generator, (**E**) a “lobster trap” for bacteria, (**F**) alternative “lobster trap” for colony of *E. coli*.

One of the major limitations of all the current SLA printers is that they are restricted to a single print material at this time. Choi et al. have developed a prototype of a multi-material SLA printer by using four different resin baths. However, the process is too complex and each resin layer requires multiple exposures, making the method quite inefficient [84]. Moreover, removing the uncured resin remains a major challenge in using this multi-material SLA for the printing of microfluidic structures. In SLA, the removal of uncured resin is easier than in i3Dp since the resin is a liquid, but it is still challenging in general [72]. There are several successful examples of using SLA for microfluidics fabrication [82], and SLA is an effective additive method for manufacturing complex nanocomposites, [73]. However, further developments of SLA are still required in order to make it an ideal method of choice for the fabrication of microfluidics for wearable biosensors [72]. Other disadvantages are the slow printing time, sometimes expensive chemicals, low biochemical adaptability of the resin, limited choice of the materials, and a resolution of a few tens of micrometers [73,83]: for example, a number of DLP printers with a resolution in XY and Z of 50 μm have been reported [73]. Even though the stereolithography process was introduced almost 33 years ago, there is still enough room for further improvements. Recently, a novel micro-diamond based composite resin was published to print a thermally conductive prototype for specific applications [72]. Of course, stereolithography is also largely used in other fields other than microfluidics (channels, valves, and pumps) for biosensing, such as organ-on-chip platforms, flexible electronics, micromixers for pKa determinations, and soft robotics [97,109]. Examples of such other systems are shown in Figure 15.

### 3.4. Multi-Material Methods

**Figure 16:**
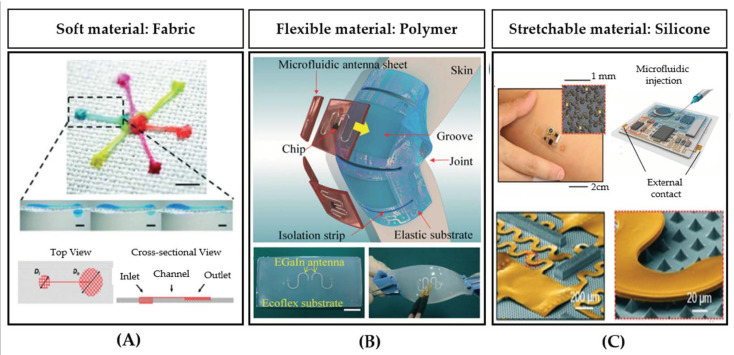
Wearable microfluidic systems realized with multi-material methods: (**A**) interfacial microfluidic transport principle to drive three-dimensional liquid flows on a micropatterned superhydrophobic textile, (**B**) triboelectric pressure sensor integrated with an antenna for data transmission, (**C**) stretchable skin-patch with integrated electronics (reprinted with permission from [116,117,118]).

As briefly mentioned at the beginning of Section 3, several multi-material methods have been proposed over the years to improve the quality of printed objects. With this approach, two or more materials can be simultaneously used for the building of a single object [87]. For example, multi-material 3D printing enables the synergic use of soft and rigid polymers with still resolutions in the range of tens of microns [27,103,115]. Several parts and components of microfluidic systems are fabricated with a multi-material method: for example, interconnects [66], membranes, valves, pumps, and multi-flow controllers [103]. All the previously discussed 3D printing methods (i3DP, FDM, and SLA) are possible with multi-materials [87,88]. The multi-material photopolymer inkjet printing method allows up to five different materials with a wide range of properties: from hard to soft plastics, elastomers, and also different colors. This method has a good speed in building an object since the multiple materials can be printed at the same time. However, with the multi-material inkjet printing method, it is difficult to remove the support material from the complex fluidic channels, while FDM printers do not need the support material to create channels. The materials used in the inkjet printing method are limited and their formulations are expensive and proprietary. The selection of the materials is often mostly concentrated on color, while the choice of flexible materials is especially suitable for the fluidics of wearable biosensors. For wearable microfluidics, three substrate interfaces are typically used, including fabric, polymer, and silicone (elastomer/rubber (see Figure 16)) [58]. These kinds of substrates are chosen since they are biocompatible and, therefore, they are particularly suitable for the biosensing systems used in wearable applications. Fabric may be soft, absorbent and breathable. Several different polymers are possible with properties of flexibility, robustness, and a strong resistance to chemicals. Silicone elastomers are stretchable and conformable, with properties of long-term durability, and present an excellent chemical resistance and viscoelasticity [58]. With multi-material methods, valves, pumps, and mixers have been demonstrated with a stronger resistance to deformation [81,87].

Most of the polymers used in multi-material additive manufacturing are split into two main categories: photosensitive polymers and thermoplastic polymers. The first group is widely used in the 3D printing method based on photopolymerization, like SLA, and includes acrylate, epoxy, or hybrid resins. SLA processes also broadly use hybrid resins, such as epoxides with acrylate content. These hybrid resins increase the integrity of the layers during the fabrication and the strength of the finished parts. More importantly, the use of hybrid resins enables the fabrication of transparent and biocompatible microfluidic devices with a high resolution. Thermoplastic polymers include acrylonitrile butadiene styrene (ABS) and polylactic acid (PLA), which are widely employed for extrusion-based methods, such as FDM [119,120]. Extruded materials for microfluidics are essentially polypropylene (PP), ABS, and PLA. PP is used for its high biocompatibility, like polydimethylsiloxane (PDMS), and it is cheaper. It is introduced as an attractive material for the additive fabrications of micro and milli-scale fluidic devices, since it is a robust, flexible, and chemically inert polymer. PP is a semi-crystalline material, which typically does not soften with raising temperatures. It quickly transforms into a low-viscosity liquid and, once solid, it shrinks less in the flow direction than in the transverse direction. PP is extruded in a liquid state, and then it solidifies via crystallization as soon as the temperature goes below the melting point. However, the thermal shrinkage stresses are high during the layer solidification, and lead to very high warping stresses. Therefore, PP is typically only used when a high biocompatibility is required. On the contrary, ABS is an amorphous polymer that can crawl slowly until it cools below the glass point, and starts to warp below the glass point to complete the solidification. Therefore, the thermal stresses above the glass point temperature could be partially compensated. Therefore, it is suitable for the building plates and chambers by minimizing the warping stress at temperatures around the glass point. ABS is a very useful material for many biomedical applications because of its excellent mechanical and processing properties, its versatility and low-cost. Most recently, one of the most common materials considered for 3D printing became PLA, which is an inexpensive, biodegradable, and nontoxic aliphatic polyester [119].

The technical limitations for multi-material printing are still present and typically related to the material’s compatibility, the adherence among them, and also the significant differences in the extrusion temperatures. In some cases, it is difficult to remove the support material and the material choice is limited with respect the typical needs in wearable biosensors. Multi-material FDM is cheaper than i3Dp, and easier than SLA. Compared to other printing methods, FDM printing has a good choice of commercially available materials with different properties. FDM typically uses thermoplastics, providing access to a wide variety of cheap and biocompatible materials. Despite these benefits, and as well as those already discussed, FDM still has its own limitations, including a low structural strength of printed objects, lack of structural integrity between the layers, and weak sealing properties when used in microfluidics for wearable biosensors. Furthermore, it provides a lower resolution with respect to SLA and i3Dp [81,87]. With multi-material SLA, the resolution and chemical compatibility is usually much better; the stretchability, gas permeability, and larger heat dissipation are better too. On the other hand, SLA is more expensive than FDM and i3Dp, and the fabrication process is typically slower because there are different resin vats that the printed object must be moved between during the printing process [81].

## 4. Printing Sensors for Direct Integration

**Figure 17:**
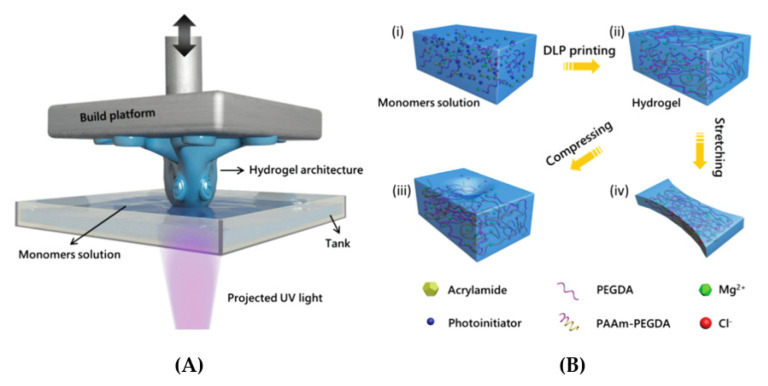
The addictive manufacture of a conductive hydrogel: (**A**) a high resolution fast bottom-up fabrication by a DLP printer, (**B**) the polymerization of a hydrogel network (reprinted with permission from [121]).

**Figure 18:**
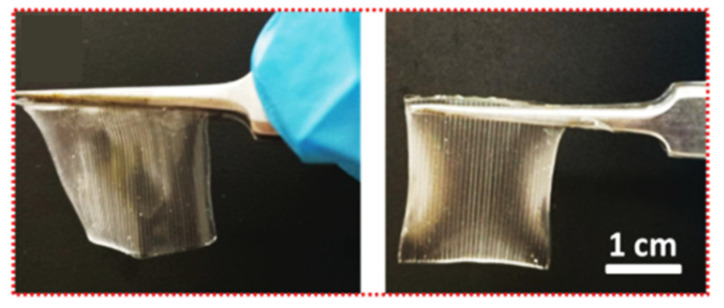
Structured hydrogel films: a total thickness of 400 mm; parallel lines with a deepness of 200 mm, line-to-line spacing of 200 mm (**in the left**) or 400 mm (**in the right** (reprinted with permission from [121])).

**Figure 19:**
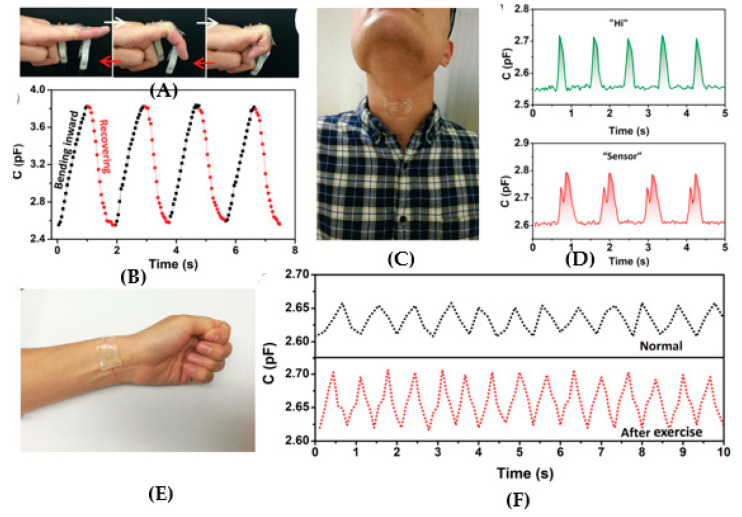
Wearable sensing devices by hydrogel films: (**A**) sensor on-the-skin for the finger bending, (**B**) capacitance as acquired from the finger cyclically bending, (**C**) sensor on-the-skin for throat movements, (**D**) acquired signals when subject says ‘‘Hi’’ and ‘‘Sensor’’, (**E**) sensor on-the-skin for the artery pulse, (**F**) radial artery pulses (reprinted with permission from [121]).

By using the additive manufacturing methods presented above, that are suitable for building the microfluidics for wearable biosensors, it is also possible to print the sensors as well for a direct integration with the fluidics. Stretchable conductors are highly suitable for wearable sensors and electronics. Typical tracks in wearable systems involve electrical conductors, such as carbon nanotubes (CNTs), graphene sheets, metal nanowires (metal NWs), liquid metals, or conductive polymers, which present limitations in these kind of applications because of an increase in resistance upon deformation. Therefore, a new class of conductors is proposed with the aim of a direct integration of sensors by direct printing into the fluidics for a wearable biosensing platform. Ionically conductive materials, mostly hydrogels (Figure 17), ionogels or polyionic elastomers, which use charged ions rather than electrons to transmit electrical signals, are suggested to achieve the aim. Many ionic conductors have the intrinsic properties of a high stretchability, transparency, and biocompatibility. More importantly, they have a good optical transmission and electrical conductance at the same time. The use of such microstructured hydrogel electrodes improves the deformability of the sensor for its application to ionic skins (ionic conductor-based sensors). Printed conductive hydrogels are highly stretchable and elastic, almost fully transparent, highly precise, and stable with regards to their electro-mechanical properties. So, they are very suitable to apply to wearable amperometric biosensors as microstructured current collectors (see Figure 18 and Figure 19). However, it is difficult to obtain a high resolution, especially in the printing of complex structures, typically due to limitations related to the low strength of the used conductive materials.

To show a good example of this kind of printed sensor, we can mention here the work of Yang et al., who proposed printing a wearable capacitive sensor for detecting both the static and dynamic pressures and strain, with a high sensitivity and low limit of detection [121]. Yang et al. fabricated an elastic and ionically conductive hydrogel by using microstructures in a single printing step with a commercial DLP printer (Figure 17A). Superior capabilities with regards to high-fidelity for the body signal acquisition over multiple skin locations was demonstrated with such a device, including finger bending tracking, pulse waveform monitoring, and larynx vibration tracking.

## 5. Conclusions

This paper provides a review of all the additive manufacturing (3D printing) methods suitable for microfluidics in applications to wearable biosensors. A photolithographic technique and chemical etching were the primary fabrication methods for microfluidics that used silicon and glass. In comparison to traditional methods, 3D printing technology has a short fabrication cycle time. This technology can have a good control of the complex design and quality of the product, with a good resolution and low-cost fabrication Wearable biosensors are required for the analysis of the molecular content of sweat as directly provided on-the-skin. Microfluidics plays an important role in solving some of the challenges for on-the-skin monitoring, for example collecting and conveying a small amount of liquid sample to the detection part. The 3D printing methods suitable for microfluidics are selective laser sintering (SLS), fused deposition modeling (FDM), inkjet 3D printing (i3Dp), laminated object manufacturing (LOM), two photon polymerization (TPP) and stereolithography (SLA). Among them, three suitable methods for flexible devices have been discussed in this paper: fused deposition modeling (FDM), inkjet 3D Printing (i3Dp), and stereolithography (SLA). These methods use polymeric substrates that have wide applications for wearable devices. On the other hand, these methods are able to use multi-material methods which have better control over the quality and flexibility of the product. Each of the discussed methods still present pros and cons when thought for fabricating microfluidics for wearable biosensors. SLA is a suitable method in case of the need for high resolutions, while SLA is not necessarily a good option for the low-cost productions or for fast fabrication processes. FDM is the more appropriate method in the case of inexpensive productions, especially in cases where biocompatibility is strictly required. The i3Dp method is most probably the best method for devices requiring extremely good mechanical properties (e.g., in term of robustness) and to assure fewer rough on-body contact-surfaces. Today, a lot of research is being done to advance these 3D printing methods for their future applications. For example, the inks in the i3Dp method can be modified for certain purposes. The biosensors required for detection on-the-skin are also printable by using additive manufacturing, especially by exploiting conductive properties of modern hydrogels, ionogels, or polyionic elastomers. Therefore, such additive manufacturing methods are definitely suitable for producing microfluidics systems with the right resolution for wearable biosensors in order to develop more advanced and fully non-invasive monitoring of metabolism directly on-the-skin of humans.

This review article will help the reader understand the importance of the role of microfluidics in the development of wearable technology and gain insight into the appropriate ways to fabricate them that will play an important role in future applications of wearable sensors using microfluidics. If a researcher wants to create suitable microfluidics for wearable sensors, by reading this article, they can decide which method is right for them according to their desires and possibilities. This article gives a brief overview of the materials used in these methods and some of their challenges. It is suggested that in future work, the efficiency, limitations, and preference of materials over each other be examined in more detail, separately for each application and method. This critical article can give researchers a good vision of the materials used in a certain method and their challenges. In this case, researchers can choose the most suitable material for the intended purpose from the available materials, and sometimes even decide to change the method by choosing a suitable material for a specific application. This study of materials and their challenges can be considered by researchers who are diligent in overcoming the challenges presented, and progress, and improve the field.

## Figures and Tables

**Table 1:** The materials, benefits, and limitations of the three 3D printing methods suitable for wearable microfluidics.

3D Printing Methods	Materials	Benefits	Drawbacks
Fused deposition modelling (FDM)	Polyethylene terephthalate (PET) Polystyrene (PS) Polycarbonate (PC) Acrylonitrile butadiene styrene (ABS) Polycaprolactone (PCL) Poly-lactic acid (PLA) Polybutylene terephthalate (PBT) Polyglycolic acid (PGA) Polypropylene(pp)	Low cost High speed Simplicity Low-cost Manufacturing of centimeter-sized prototypes Using inexpensive biocompatible polymers	Weak mechanical properties Limited materials (only thermoplastics) Layer-by-layer finish Leakage due to filament bonding Difficulty of removal of support structure for complex internal features Inter-layer distortion
Inkjet printing (i3Dp)	Soft elastomers Liquid metals (i.e., EGaIn) Wax-based inks Liquid suspensions Acrylonitrile butadiene styrene (ABS), Polystyrene (PS), Polypropylene (PP), Polymethylmethacrylate (PMMA), Polycarbonate (PC) Ethylene propylene diene monomer (EPDM) High-impact polystyrene (HIPS)	Layer-by-layer fine structures Fast High resolution smooth surface Low cost Ability to easily print highly complex devices without using lithography Precise control Realizing microfluidics directly on other systems without any bonding steps Absence of sticking agents in between layers	Difficulty in removing the support material Layer-by-layer finish
Stereolithography (SLA)	Epoxy Hybrid resins Acrylate based resin Clear acrylic polymer Elastomers and ceramics Composites of photopolymers Hybrid polymer-ceramics	High quality Smooth surface Use flexible resin Fine resolution (a nometer scale) custom low-cost resins No need for external alignment Ability to directly print the channels Manufacturing complex nanocomposites Making a monolithic structure without the need for bonding	Slow printing Sometimes expensive chemicals Low biochemical adaptability of the resist Limited choice of the materials
